# Molecular epidemiology of drug resistance markers of *Plasmodium falciparum* in Yunnan Province, China

**DOI:** 10.1186/1475-2875-11-243

**Published:** 2012-07-28

**Authors:** Fang Huang, Linhua Tang, Henglin Yang, Shuisen Zhou, Hui Liu, Junwei Li, Shaohua Guo

**Affiliations:** 1 National Institute of Parasitic Diseases, Chinese Center for Disease Control and Prevention, WHO Collaborating Centre for Malaria, Schistosomiasis and Filariasis, Key Laboratory of Parasite and Vector Biology, Ministry of Health, Shanghai, 200025, PR China; 2 Yunnan Institute of Parasitic Diseases, Puer, 665000, PR China

## Abstract

**Background:**

The mutations in *Plasmodium falciparum* chloroquine resistance transporter (*pfcrt*), multidrug resistance 1 (*pfmdr1*), dihydrofolate reductase (*pfdhfr*), dihydropteroate synthase (*pfdhps*) and ATPase (*pfatp6*) genes were associated with anti-malaria drug resistance. The aim of this study was to investigate the prevalence of polymorphisms in *pfcrt*, *pfmdr1*, *pfdhfr*, *pfdhps* and *pfatp6* in Yunnan Province. Finger-prick blood samples were collected from malaria-positive patients from Yunnan Province in 2009-2010. Single-nucleotide polymorphisms (SNPs) in the resistance-related genes were analysed by various PCR-based methods.

**Results:**

A total of 108 blood samples were collected. Although chloroquine has not been used to treat falciparum malaria for nearly 30 years, 95.3% of the parasites still carried the *pfcrt* K76T mutation, whereas the majority of isolates displayed the wild-type *pfmdr1* N86 and D1246 sequences. The molecular level of sulphadoxine–pyrimethamine resistance in *P. falciparum* was high. The most prevalent mutation was *pfdhfr* C59R (95.9%), whereas the frequencies of the quadruple, triple and double mutants were 22.7% (N51I/C59R/S108N/I164L), 51.5% (N51I/C59R/S108N, N51I/C59R/I164L and C59R/S108N/ I164L) and 21.6% (N51I/ C59R, C59R/S108N and C59R/I164L), respectively. A437G (n = 77) and K540E (n = 71) were the most prevalent mutations in *pfdhps*, and 52.7% of the samples were double mutants, among which A437G/K540E was the most common double mutation (37/49). Quadruple mutants were found in 28.0% (26/93) of samples. A total of 8.6% of isolates (8/93) carried the S436A/A437G/A581G triple mutation. No mutations were found in *pfatp6* codons 623 or 769, but another two mutations (N683K and R756K) were found in 4.6% (3/97) and 9.2% (6/97) of parasite isolates, respectively.

**Conclusions:**

This study identified a high frequency of mutations in *pfcrt*, *pfdhfr* and *pfdhps* associated with CQ and SP resistance in *P. falciparum* and no mutations linked to artemisinin resistance (*pfatp6*). Molecular epidemiology should be included in routine surveillance protocols and used to provide complementary information to assess the appropriateness of the current national anti-malarial drug policy.

## Background

Malaria is a major health problem in Southeast Asia, where 1.3 billion people are at risk, and it causes approximately 120,000 deaths each year [1]. Despite significant reductions in the overall malaria burden in the 20th century, the disease still represents a significant public health problem in China, especially in Yunnan Province [2]. In 2010, the Ministry of Health in China launched an “action plan for malaria elimination”, with the goals of eradicating local malaria cases in regions outside of the Yunnan border area by the end of 2015 and eliminating malaria in the entire country by the end of 2020 [3].

Falciparum malaria is now found in only two provinces in China, the Yunnan and Hainan Provinces [4]. Malaria control measures have been actively implemented for more than 30 years, and considerable success has been achieved; there have been no local cases of falciparum malaria in Hainan in the past two years [5]. However, the malaria situation in Yunnan Province near the Myanmar border remains serious. Yunnan Province is located in southern China; it includes 131 counties and borders Myanmar, Lao People’s Democratic Republic and Vietnam. The majority of malaria cases are concentrated in several counties bordering Myanmar. The border with Myanmar is 1,997 km long and approximately 98 million individuals cross the border each year. Yunnan Province has an estimated population of 42.36 million, with about 35.52 million people residing in malaria-endemic areas. From 2005 to 2011, malaria incidence decreased across the province and the reported cases and deaths in the province population have declined from 13,239 cases and 37 deaths in 2005 to 1,216 cases and zero deaths in 2011. Of 2,643 reported cases, 28% were caused by *Plasmodium falciparum*[6].

The first case of chloroquine (CQ) resistance in *P. falciparum* was found in Yunnan Province in 1973, and by the end of the 1970s, CQ resistance had spread widely [7-9]. Subsequently, sulphadoxine–pyrimethamine (SP) was introduced as the first-line drug for falciparum malaria treatment. This antifolate combination seemed to be an effective and reasonable alternative, but resistance to SP also developed soon after its introduction in China. Since 2001, the WHO has recommended the use of artemisinin-based combination therapy in all areas where *P. falciparum* is resistant to other anti-malarial medicines to optimize therapeutic effectiveness and delay the emergence of resistance. The WHO advocated a complete ban on artemisinin monotherapy for uncomplicated malaria in 2006 [10]. The national drug policy of China was updated in 2009, and the first-line drugs currently used to treat falciparum malaria is artemisinin-based combination therapy (ACT), which includes dihydroartemisinin-piperaquine (DHA-PIP), artesunate + amodiaquine, artemisinin-naphthoquine phosphate and artemisinin-piperaquine [11].

Many factors have contributed to the development and spread of drug resistance, including gene mutations and drug pressure [12]. Molecular surveillance is a new technique that has been implemented over the past decades to provide complementary information to assess the appropriateness of current policies based on anti-malarial drugs [13]. Several molecular markers of *P. falciparum* resistance have been identified. The K76T allele in the CQ resistance transporter gene (*pfcrt*) is associated with CQ and amodiaquine treatment failure and could be used for the surveillance of clinical CQ resistance [14-16]. The Y86 allele of multidrug resistance gene 1 (*pfmdr1*) has been linked with CQ and amodiaquine resistance [17]. The role of dihydrofolate reductase (*dhfr*) and dihydropteroate synthase (*dhps*) mutations in the mechanism of resistance to SP drugs has been well described. Mutations associated with antifolate resistance have been identified in codons 436, 437, 540, 581 and 613 in the *pfdhps* gene and codons 108, 51, 59, 140, 16 and 164 in the *pfdhfr* gene. The quintuple mutant of *pfdhfr* (S108N, N51I and C59R) and *pfdhps* (A437G and K540E) were associated with a high relative risk of treatment failure, and this haplotype was suggested as a relevant molecular marker for failure of SP treatment in uncomplicated *P. falciparum* cases [18-21]. The sarco/endoplasmic reticulum Ca^2+^-ATPase ortholog of *P. falciparum* (*pfatp6*) was suggested to be involved in the mechanism of parasite resistance to artemisinin [22,23]. Specific point mutations in codons 769, 623 and 431 were associated with artemisinin resistance [24].

In recent decades, malaria drug resistance surveillance in Yunnan Province has relied on *in vitro* and *in vivo* tests, whereas molecular epidemiological studies of drug resistance have only been conducted as limited studies [25,26] and have not been used as a routine surveillance tool in the national malaria programme. In this study, the prevalence of polymorphisms in *pfcrtpfmdr1pfdhfrpfdhps* and *pfatp6* genes in blood samples obtained from *P. falciparum-*infected patients in Yunnan Province were determined.

## Methods

### Sample collection and DNA extraction

The study was reviewed and approved by the ethics committee of the Chinese Centre for Disease Control and Prevention (China CDC). Blood samples were collected from patients with uncomplicated *P. falciparum* infection prior to drug treatment. All of the patients came from township hospitals in Tengchong and Yingjiang Counties in year 2009-2010. The initial diagnosis was made by microscopic examination of Giemsa-stained thick blood films or a rapid diagnostic test. For each sample, approximately 200 μl of finger-prick blood was spotted on a piece of Whatman 3M 903 filter paper and air dried. After the patients were confirmed as malaria, they would get DHA-PIP. The dried filters were stored in individual plastic bags at -20°C until DNA extraction. Parasite DNA was extracted from the blood filters using a QIAamp DNA mini kit (Qiagen, Valencia, CA, USA).

### Genetic characterization of parasites

Nested PCR [14,20,27] was used to amplify fragments of *pfcrtpfmdr1pfdhfrpfdhps* and *pfatp6*. The amplified products were purified from an agarose gel and sequenced with an automated DNA sequencer (ABI systems, Perkin-Elmer, France). Sequence alignments and analysis were carried out using Mega and BioEdit software. Amino acid sequences were compared with wild-type sequences. The sequences of the amplicons were aligned with 3D7 strain published data from the NCBI database by BLAST analysis.

## Results

### Study sites

**Figure 1:**
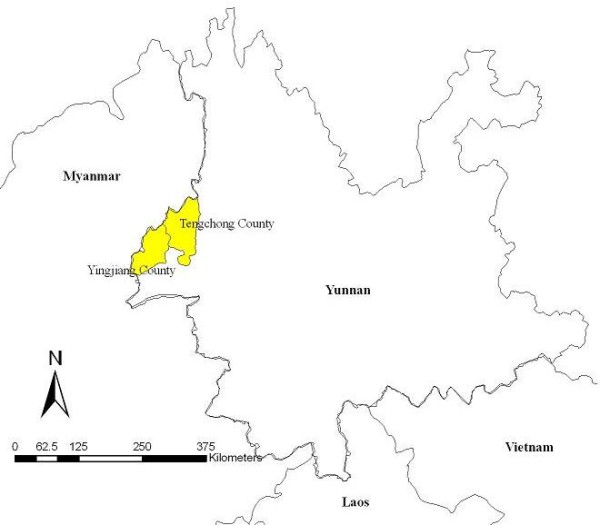
The location of Yingjiang and Tengchong Counties relative to neighbouring countries.

Yingjiang and Tengchong Counties, which have the highest falciparum malaria incidence rates in China, are located in the Dehong and Baoshan Prefectures, respectively, in southwest Yunnan Province (Figure 1). In this study, four township hospitals bordering with Myanmar were selected to collect malaria samples. Of all of samples, 56 patients was local malaria cases and 52 patients were contracted from Kachin State of Myanmar.

### Prevalence of single-nucleotide polymorphisms in *pfcrt*

Codons 71, 72 and 76 were successfully amplified in 106 of the 108 samples analysed. The mutation in *pfcrt* codon 76 was found in 95.3% (101/106) of parasite isolates, and mutations in codons 71 and 72 were found in 3.0% (3/101) of parasite isolates. The mutant haplotype CVIET in codons 72-76 was identified in 3% (3/101) of samples and 5.0% (5/101) contained the wild-type haplotype CVMNK.

### Prevalence of single-nucleotide polymorphisms in *pfmdr1*

All of the samples were genotyped for *pfmdr1* at codons 86 and 1246. The majority of isolates displayed the wild-type *pfmdr1* N86 and D1246 alleles. A total of 5.6% (6/108) of isolates carried the mutant allele Y86, and 94.4% had the wild-type allele. No mutation in Y1246 was found.

### Prevalence in single-nucleotide polymorphisms in *pfdhfr* and *pfdhps*

The single-nucleotide polymorphism (SNP) haplotype of the *pfdhfr* gene at codons 16, 51, 59, 108, 140 and 164 is linked with *P. falciparum* pyrimethamine resistance. The *pfdhfr* gene was successfully amplified in 97 samples, four of which contained amino acid substitutions compared with the wild-type sequence. The most prevalent mutation was C59R (95.9%). There were no mutations in codons 16 or 140. A total of 38 of 97 isolates carried the wild-type N51 allele, while 55 carried the mutant I51 allele, and four had mixed alleles; 93 and 58 of 97 isolates had mutant R59 and N108, respectively. A total of 18.6% (18/97) of isolates carried the wild-type I164, while 75.3% (73/97) carried mutant L164, and 6 had mixed alleles (Table 1).

**Table 1:** **Prevalence of haplotypes of SNPs in*pfdhfr*and*pfdhps*in*P. falciparum*isolates from Yunnan Province**

**Gene**	**Haplotypes**	**N**	**%**
***pfdhfr***	I51	59	72.3
	R59	93	95.1
	N108	96	94.1
	L164	79	85.3
***pfdhps***	I51/R59/N108	35	34.7
	A436	41	59.7
	G437	77	94.0
	E540	71	69.0
	G581	50	69.5
	G437/ E540	37	38.1
***pfdhfr/pfdhps***	I51/R59/N108+ G437/ E540	19	19.8

**Figure 2:**
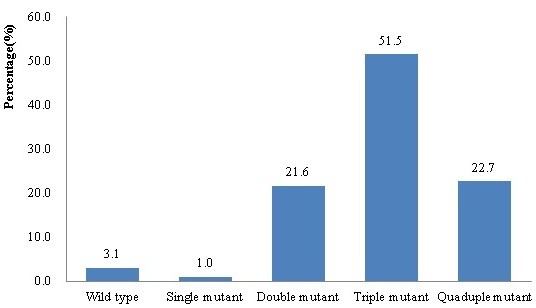
**Frequency of*pfdhfr*haplotypes in samples from Yingjiang County.** The frequency of the constructed haplotypes of SNPs in N51I, C59R, S108N and I164L of the *pfdhfr* gene is linked with *Plasmodium falciparum* pyrimethamine resistance (n = 97).

More than half (51.5%) of the isolates had triple mutations, including N51I/C59R/S108N (n = 35), N51I/C59R/I164L (n = 20) and C59R/S108N/ I164L (n = 21). The double mutants included N51I/ C59R (n = 5), C59R/S108N (n = 6) and C59R/I164L (n = 10). A total of 22.7% (22/97) carried quadruple mutations at codons N51I/ C59R/S108N/I164L, and only one isolate had a single mutation at codon R59 (Figure 2).

A total of 93 parasite samples were genotyped for *pfdhps* polymorphisms at codons 436, 437, 540, 581 and 613, which are associated with *P. falciparum* sulphadoxine resistance. The A437G (n = 77) and K540E (n = 71) mutants were most prevalent in *pfdhps*. A total of 52 isolates carried the wild-type S436 allele, and 41 had the mutant A436 allele. A total of 53.8% of samples carried the wild-type A581 allele, while 44.1% (50/93) had the G581 allele, and 2 isolates had mixed alleles (Table 1).

**Figure 3:**
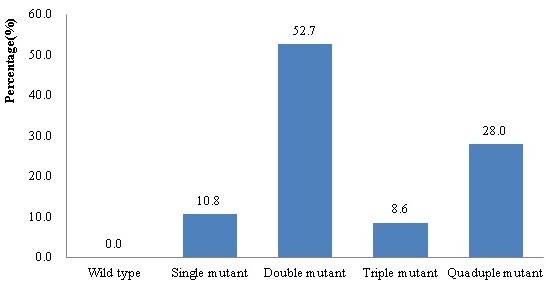
**Frequency of*pfdhps*haplotypes in samples from Yingjiang County.** The frequency of the constructed SNP haplotypes in the *pfdhps* gene at S436A, A437G, K540E and A581G is linked with *Plasmodium falciparum* sulphadoxine resistance (n = 93).

Double *pfdhps* mutants were found in 52.7% of isolates (49/93). The most prevalent double mutant was A437G/K540E (N = 37), followed by S436A/A437G (n = 4), S436A//K540E (n = 1), A437G/A581G (n = 3) and K540E/A581G (n = 4). Quadruple mutants were found in 28.0% (26/93) of samples. A total of 8.6% (8/93) of samples were S436A/A437G/A581G triple mutants, and 10 isolates had a single mutation at A436 (n = 4), E540 (n = 5) or G581 (n = 1) (Figure 3).

Besides, the quintuple mutant of *pfdhfr* (S108N, N51I and C59R) and *pfdhps* (A437G and K540E) were shown in Table 1, which werewere associated with a high relative risk of treatment failure, and this haplotype was suggested as a relevant molecular marker for SP resistance.

### Prevalence of single-nucleotide polymorphisms in *pfatp6*

No mutations in *pfatp6* codons 623 or 769 were found, but another two mutations (N683K and R756K) were found in 4.6% (3/97) and 9.2% (6/97) of parasite isolates, respectively.

## Discussion

Yunnan Province has experienced considerable economic development in the last 10 years; based on the appearance of villages at present compared with 1999-2000, the standard of living, even in rural areas, has improved considerably. During this time, there has also been a significant decline in the number of malaria cases reported. However, suspected artemisinin resistance occurring in Mekong areas and difficulties for mobile population management in bordering areas will presents a challenge for eliminating malaria in the entire country by the end of 2020.

*Pfcrt* has been demonstrated to be a major determinant of CQ resistance in *P. falciparum*, and the K76T mutation has been widely used as a reliable marker for CQ resistance in epidemiological studies [28-30]. Since CQ resistance was first found in China, it has spread widely, prompting the use of ACT to treat falciparum malaria. According to a longitudinal survey conducted in the Yunnan and Hainan Provinces, which are endemic for falciparum malaria, the resistance of *P. falciparum* to CQ declined progressively after CQ use was stopped or reduced, as determined by *in vitro* tests [9,31]. However, the result of this study showed a high frequency of the K76T mutation of *pfcrt*, which is consistent with other studies conducted in China [25,26]. Several theories have been proposed for the persistence of the K76T mutation. The most important proposed reason was the wide use of CQ to treat *Plasmodium vivax*. Although CQ has not been used to treat *P. falciparum* in China for more than 30 years, the stable and high prevalence of CQ resistance may be caused by the continued use of CQ as a first-line drug for the treatment of *P. vivax*. This factor may be responsible for the slow decline of *P. falciparum* CQ resistance in Southeast Asia, especially in the Mekong sub-region [32,33].

Compared with the high prevalence of the *pfcrt* mutation, few samples had mutations in codon 86 and no mutations were found in codon 1246 of *pfmdr1*. This result was consistent with the findings of other researchers [25,26,34,35]. The Y1246 allele of *pfmdr1* was not related with CQ resistance, which was not consistent with the results in Africa [36]. However, the prevalence of the mutation at Y86 was still high in parasite isolates from some countries in Southeast Asia. *Pfmdr1* mutations differentially affected the CQ responses in CQ resistance parasites and their activities depending on the *pfcrt* haplotype to which they were associated [37,38], and *pfmdr1* gene copy number amplifications and most of the *pfmdr1* gene amplifications in field samples harbors an asparagine at 86 amino acid position of *pfmdr1* gene [39,40]. Mefloquine has never been used in Yunnan Province, which would also be related to the low prevalence of mutation of the *pfmdr1* gene.

The role of *pfdhfr* and *pfdhps* mutations in the mechanism of resistance to SP drugs has been well described. Pyrimethamine was used for the radical treatment of *P. vivax* in combination with primaquine 40 years ago. Additionally, pyrimethamine added to salt was used for prophylaxis in the 1980s [30]. Pyrimethamine plus primaquine has always been recommended as prophylactic medicine for specific populations in China [6]. This study found that the prevalence of quadruple, triple and double mutant *P. falciparum* at the China-Myanmar border was still high. The finding of this study was similar to that in northeast Myanmar, Thailand and Cambodia, where highly mutated *pfdhfr* and *pfdhps* genotypes were also common [41,42]. SP monotherapy was used for a short time after CQ resistance had spread widely, and artemisinin drugs were subsequently introduced and gradually became the standard of malaria therapy. However, it is unclear how to explain the discrepancy between the absence of SP pressure and the high prevalence of the *pfdhfr* triple mutants and *pfdhps* double mutants. it could be speculated that SP drugs were frequently used in bordering countries of the Mekong sub-region for long periods, and the frequent population migration across borders ensured high regional gene flow, including these drug resistance genes.

The mechanism of action of artemisinin remains controversial.In vivo artemisinin resistance has been proposed [43] and identified by the presence of significantly decreased parasite reduction rates, manifested clinically by markedly longer parasite clearance times from the body [44-47]. The molecular basis for this phenomenon is uncertain. One of the proposed mechanisms is the interaction with the sarcoplasmic reticulum Ca^2+^ ATPase6. The analysis of naturally occurring polymorphisms in *pfatp6* in field isolates suggested that a polymorphism at codon 769 may be associated with the reduced susceptibility of these isolates to artemether *in vitro*[23]. Like other investigators, there were no polymorphisms in codons 263 and 769, described as the key amino acids for the interaction between *pfatp6* and artemisinin [24]. However, two mutations (N683K and R756K) were identified in parasite isolates in this study, which has been found in Zanzibar and Tanzania [48], and no mutation in position 683 has been published previously [49]. Mutations observed in this study and previous studies [50,51] could be indirectly implicated in artemisinin susceptibility. Although artemisinin resistance in western Cambodia seems to be a heritable genetic trait, none of the candidate genes suggested by earlier studies confer artemisinin resistance. A genome-wide approach using whole genome sequencing and transcriptome studies to identify the molecular basis of artemisinin resistance has been suggested [52].

## Conclusion

This study reports the high frequency of mutations in *pfcrt*, *pfdhfr* and *pfdhps,* which are associated with CQ and SP resistance in *P. falciparum,* but no mutation linked with artemisinin resistance in *pfatp6*. Although, molecular marker of artemisinin resistance has not been identified yet, this result of other *in vivo* tests in Yunnan Province (unpublished data) indicates that current ACT drugs for *P. falciparum* treatment are still effective. In addition, molecular epidemiology should be part of routine surveillance to produce complementary information to assess the appropriateness of the current national anti-malarial drug policy.

## Competing interests

The authors hereby certify that no conflict of interest of any kind occurred in the framework of this study.

## Authors’ contributions

FH was responsible for the molecular genetic analysis and data interpretation and drafted the manuscript. LT was responsible for the overall study and was involved in all stages of this study, including its design. HY was involved in the study design and coordination. SZ performed several of the molecular genetic studies and data analysis. HL participated in sample collection. JL and SG carried out the molecular analysis and sequence alignments. All the authors read and approved the final manuscript.

## References

[B1] Malaria Disease Burden in SEA Region [http://www.searo.who.int/EN/Section10/Section21/Section340_4018.htm]

[B2] Tang LH Progress in malaria control in China Chin Med J 2000 115 69 92 11775219

[B3] China action plan for malaria eliminationhttp://www.moh.gov.cn/publicfiles/business/htmlfiles/mohjbyfkzj/s3593/201005/47529.htm

[B4] Zhou SS Wang Y Fang W Tang LH Malaria situation in the People’s Republic of China in 2008 Chin J Parasitol Parasit Dis 2009 27 455 457 20232622

[B5] Zhou SS Wang Y Li Y Malaria situation in the People’s Republic of China in 2010 Chin J Parasitol Parasit Dis 2011 29 401 403 24822335

[B6] Malaria Surveillance Project in China 2005 Ministry of Health, Beijing

[B7] Liu DQ Liu RJ Ren DX Gao DQ Zhang CY Qiu CP Cai XZ Ling CF Liang AH Tang Y Alteration in resistance of Plasmodium falciparum to chloroquine after cessation of chloroquine medication for twelve years Zhongguo Ji Sheng Chong Xue Yu Ji Sheng Chong Bing Za Zhi 1992 10 241 244 1303327

[B8] Liu DQ Liu RJ Ren DX Gao DQ Zhang CY Qiu CP Cai XZ Ling CF Liang AH Tang Y Changes in the resistance of Plasmodium falciparum to chloroquine in Hainan, China Bull World Health Organ 1995 73 483 486 7554020 PMC2486789

[B9] Liu DQ Feng XP Yangh HL Lin SG Chen WJ Yang PF Fluctuation in the Resistance of Plasmodium falciparum to chloroquine in China Chin J Parasitol Parasit Dis 2005 23 27 31 16042203

[B10] WHO Guidelines for the treatment of malaria 2010 World Health Organization, Geneva 25473692

[B11] Antimalarial drug policy in Chinahttp://www.moh.gov.cn/publicfiles/business/htmlfiles/mohjbyfkzj/s3594/200907/41610.htm

[B12] Talisuna AO Bloland P D’Alessandro U History, dynamics, and public health importance of malaria parasite resistance Clin Microbiol Rev 2004 17 235 254 10.1128/CMR.17.1.235-254.2004 14726463 PMC321461

[B13] WHO Methods for surveillance of antimalarial drug efficacy 2009 World Health Organization, Geneva

[B14] Djimde A Doumbo OK Cortese JF Kayentao K Doumbo S Diourte Y Dicko A Su XZ Nomura T Fidock DA Wellems TE Plowe CV Coulibaly D A molecular marker for chloroquine-resistant falciparum malaria N Engl J Med 2001 344 257 263 10.1056/NEJM200101253440403 11172152

[B15] Ursing J Kofoed PE Rodrigues A Rombo L Gil JP Plasmodium falciparum genotypes associated with chloroquine and amodiaquine resistance in Guinea-Bissau Am J Trop Med Hyg 2007 76 844 848 17488902

[B16] Picot S Olliaro P de Monbrison F Bienvenu AL Price RN Ringwald P A systematic review and meta-analysis of evidence for correlation between molecular markers of parasite resistance and treatment outcome in falciparum malaria Malar J 2009 8 89 10.1186/1475-2875-8-89 19413906 PMC2681474

[B17] Babiker HA Pringle SJ Abdel-Muhsin A Mackinnon M Hunt P Walliker D High-level chloroquine resistance in Sudanese isolates of Plasmodium falciparum is associated with mutations in the chloroquine resistance transporter gene pfcrt and the multidrug resistance Gene pfmdr1 J Infect Dis 2001 183 1535 1538 10.1086/320195 11319692

[B18] Basco LK Ringwald P Molecular epidemiology of malaria in Yaounde, Cameroon. VI. Sequence variations in the Plasmodium falciparum dihydrofolate reductase-thymidylate synthase gene and in vitro resistance to pyrimethamine and cycloguanil Am J Trop Med Hyg 2000 62 271 276 10813484 10.4269/ajtmh.2000.62.271

[B19] Hyde JE Drug-resistant malaria - an insight FEBS J 2007 274 4688 4698 10.1111/j.1742-4658.2007.05999.x 17824955 PMC2720519

[B20] Kublin JG Dzinjalamala FK Kamwendo DD Malkin EM Cortese JF Martino LM Mukadam RA Rogerson SJ Lescano AG Molyneux ME Winstanley PA Chimpeni P Taylor TE Plowe CV Molecular markers for failure of sulfadoxine-pyrimethamine and chlorproguanil-dapsone treatment of Plasmodium falciparum malaria J Infect Dis 2002 185 380 388 10.1086/338566 11807721

[B21] Lynch C Pearce R Pota H Cox J Abeku TA Rwakimari J Naidoo I Tibenderana J Roper C Emergence of a dhfr mutation conferring high level drug resistance in Plasmodium falciparum populations from southwest Uganda J Infect Dis 2008 197 1598 1604 10.1086/587845 18471065

[B22] Eckstein-Ludwig U Webb RJ Van Goethem ID East JM Lee AG Kimura M O'Neill PM Bray PG Ward SA Krishna S Artemisinins target the SERCA of Plasmodium falciparum Nature 2003 424 957 961 10.1038/nature01813 12931192

[B23] Uhlemann AC Cameron A Eckstein-Ludwig U Fischbarg J Iserovich P Zuniga FA East M Lee A Brady L Haynes RK Krishna S A single amino acid residue can determine the sensitivity of SERCAs to artemisinins Nat Struct Mol Biol 2005 12 628 9 10.1038/nsmb947 15937493

[B24] Jambou R Legrand E Niang M Khim N Lim P Volney B Ekala MT Bouchier C Esterre P Fandeur T Mercereau-Puijalon OS Resistance ofPlasmodium falciparumfield isolates to in-vitro artemether and point mutations of the SERCA-type PfATPase6 Lancet 2005 366 1960 1963 10.1016/S0140-6736(05)67787-2 16325698

[B25] Yang Z Zhang Z Sun X Wan W Cui L Molecular analysis of chloroquine resistance in Plasmodium falciparum in Yunnan Province China Trop Med Int Health 2007 12 1051 60 10.1111/j.1365-3156.2007.01882.x 17875016

[B26] Zhang GQ Guan YY Zheng B Wu S Tang LH Molecular assessment of Plasmodium falciparum resistance to antimalarial drugs in China Trop Med Int Health 2009 14 1266 71 10.1111/j.1365-3156.2009.02342.x 19772548

[B27] WHO Methods and techniques for clinical trials on antimalarial drug efficacy: genotyping to identify parasite populations 2008 World Health Organization, Geneva http://www.who.int/malaria/resistance

[B28] Fidock DA Nomura T Talley AK Cooper RA Dzekunov SM Ferdig MT Ursos LM Sidhu AB Naudé B Deitsch KW Su XZ Wootton JC Roepe PD Wellems TE Mutations in the P. falciparum digestive vacuole transmembrane protein PfCRT and evidence for their role in chloroquine resistance Mol Cell 2000 6 861 871 10.1016/S1097-2765(05)00077-8 11090624 PMC2944663

[B29] Lakshmanan V Bray PG Verdier-Pinard D Johnson DJ Horrocks P Muhle RA Alakpa GE Hughes RH Ward SA Krogstad DJ Sidhu AB Fidock DA A critical role for PfCRT K76T in Plasmodium falciparum verapamil-reversible chloroquine resistance EMBO J 2005 24 2294 2305 10.1038/sj.emboj.7600681 15944738 PMC1173140

[B30] Wang RZ Efficiency of pyrimethamine salt for P. vivax prophylaxis Railway Medicine 1981 4 246 247

[B31] Yang HL Yang PF Dong Y Che LG Chen WC He H Liu DQ Liu RJ Zhan B Zhang CY Gao DQ Longitudinal surveillance of chloroquine resistance of Plasmodium falciparum after cessation of medication in south Yunnan Zhongguo Ji Sheng Chong Xue Yu Ji Sheng Chong Bing Za Zhi 1994 12 31 33 8044901

[B32] Durrand V Berry A Sem R Glaziou P Beaudou J Fandeur T Variations in the sequence and expression of the Plasmodium falciparum chloroquine resistance transporter (Pfcrt) and their relationship to chloroquine resistance in vitro Mol Biochem Parasitol 2004 136 273 85 10.1016/j.molbiopara.2004.03.016 15478806

[B33] Congpuong K Na Bangchang K Mungthin M Bualombai P Wernsdorfer WH Molecular epidemiology of drug resistance markers of Plasmodium falciparum malaria in Thailand Trop Med Int Health 2005 10 717 722 10.1111/j.1365-3156.2005.01450.x 16045457

[B34] Guan YY Tang LH Hu L Feng XP Liu DQ The point mutations in Pfcrt and Pfmdr1 genes in Plasmodium falciparum isolated from Hainan Province Chin J Parasitol Parasit Dis 2005 23 135 139 16299999

[B35] Duraisingh MT Jones P Sambou I von Seidlein L Pinder M Warhurst DC The tyrosine-86 allele of the pfmdr1 gene of Plasmodium falciparum is associated with increased sensitivity to the anti-malarials mefloquine and artemisinin Mol Biochem Parasitol 2000 108 13 23 10.1016/S0166-6851(00)00201-2 10802315

[B36] Foote SJ Kyle DE Martin RK Oduola AM Forsyth K Kemp DJ Cowman AF Several alleles of the multidrug-resistance gene are closely linked to chloroquine resistance in Plasmodium falciparum Nature 1990 345 255 25 10.1038/345255a0 2185424

[B37] Patel JJ Thacker D Tan JC Pleeter P Checkley L Gonzales JM Deng B Roepe PD Cooper RA Ferdig MT Chloroquine susceptibility and reversibility in a Plasmodium falciparum genetic cross Mol Microbiol 2010 78 770 787 10.1111/j.1365-2958.2010.07366.x 20807203 PMC3091165

[B38] Sá JM Twu O Hayton K Reyes S Fay MP Ringwald P Wellems TE Geographic patterns of Plasmodium falciparum drug resistance distinguished by differential responses to amodiaquine and chloroquine Proc Natl Acad Sci USA 2009 106 18883 18889 10.1073/pnas.0911317106 19884511 PMC2771746

[B39] Price RN Uhlemann AC Brockman A McGready R Ashley E Phaipun L Patel R Laing K Looareesuwan S White NJ Nosten F Krishna S Mefloquine resistance in Plasmodium falciparum and increased pfmdr1 gene copy number Lancet 2004 364 438 447 10.1016/S0140-6736(04)16767-6 15288742 PMC4337987

[B40] Veiga MI Ferreira PE Jörnhagen L Malmberg M Kone A Schmidt BA Petzold M Björkman A Nosten F Gil JP Novel polymorphisms in Plasmodium falciparum ABC transporter genes are associated with major ACT antimalarial drug resistance PLoS One 2011 6 20212 10.1371/journal.pone.0020212 PMC3102103 21633513

[B41] Khim N Bouchier C Ekala MT Incardona S Lim P Legrand E Jambou R Doung S Puijalon OM Fandeur T Countrywide survey shows very high prevalence of Plasmodium falciparum multilocus resistance genotypes in Cambodia Antimicrob Agents Chemother 2005 49 3147 3152 10.1128/AAC.49.8.3147-3152.2005 16048916 PMC1196218

[B42] Anderson TJ Nair S Sudimack D Williams JT Mayxay M Newton PN Guthmann JP Smithuis FM Tran TH van den Broek IV White NJ Nosten F Geographical distribution of selected and putatively neutral SNPs in Southeast Asian malaria parasites Mol Biol Evol 2005 22 2362 2374 10.1093/molbev/msi235 16093566

[B43] Noedl H Artemisinin resistance: how can we find it? Trends Parasitol 2005 21 404 405 10.1016/j.pt.2005.06.012 16046187

[B44] Dondorp AM Nosten F Yi P Das D Phyo AP Tarning J Lwin KM Ariey F Hanpithakpong W Lee SJ Ringwald P Silamut K Imwong M Chotivanich K Lim P Herdman T An SS Yeung S Singhasivanon P Day NP Lindegardh N Socheat D White NJ Artemisinin resistance in Plasmodium falciparum malaria N Engl J Med 2009 361 455 467 10.1056/NEJMoa0808859 19641202 PMC3495232

[B45] Noedl H Se Y Schaecher K Smith BL Socheat D Fukuda MM Evidence of artemisinin-resistant malaria in western Cambodia N Engl J Med 2008 359 2619 2620 10.1056/NEJMc0805011 19064625

[B46] Noedl H Se Y Sriwichai S Schaecher K Teja-Isavadharm P Smith B Rutvisuttinunt W Bethell D Surasri S Fukuda MM Socheat D Chan Thap L Artemisinin resistance in Cambodia: a clinical trial designed to address an emerging problem in Southeast Asia Clin Infect Dis 2010 51 82 89 10.1086/657120 21028985

[B47] Dahlström S Veiga MI Ferreira P Mårtensson A Kaneko A Andersson B Björkman A Gil JP Diversity of the sarco/endoplasmic reticulum Ca(2+)-ATPase orthologue of Plasmodium falciparum (PfATP6) Infect Genet Evol 2008 8 340 345 10.1016/j.meegid.2008.02.002 18359278

[B48] Bertaux L le Quang H Sinou V Thanh NX Parzy D New PfATP6 mutations found in Plasmodium falciparum isolates from Vietnam Antimicrob Agents Chemother 2009 53 4570 4571 10.1128/AAC.00684-09 19687249 PMC2764200

[B49] Dahlström S Veiga MI Ferreira P Mårtensson A Kaneko A Andersson B Björkman A Gil JP Diversity of the sarco/endoplasmic reticulum Ca2 + -ATPase orthologue of Plasmodium falciparum (PfATP6) Infect Genet Evol 2008 8 340 345 10.1016/j.meegid.2008.02.002 18359278

[B50] Ibrahim ML Khim N Adam HH Ariey F Duchemin JB Polymorphism of PfATPase in Niger: detection of three new point mutations Malar J 2009 8 28 10.1186/1475-2875-8-28 19226462 PMC2661090

[B51] Menegon M Sannella AR Majori G Severini C Detection of novel point mutations in the Plasmodium falciparum ATPase6 candidate gene for resistance to artemisinins Parasitol Int 2008 57 233 235 10.1016/j.parint.2007.12.004 18187359

[B52] Imwong M Dondorp AM Nosten F Yi P Mungthin M Hanchana S Das D Phyo AP Lwin KM Pukrittayakamee S Lee SJ Saisung S Koecharoen K Nguon C Day NP Socheat D White NJ Exploring the contribution of candidate genes to artemisinin resistance in Plasmodium falciparum Antimicrob Agents Chemother 2010 54 2886 2892 10.1128/AAC.00032-10 20421395 PMC2897287

